# A Novel Radar HRRP Recognition Method with Accelerated T-Distributed Stochastic Neighbor Embedding and Density-Based Clustering

**DOI:** 10.3390/s19235112

**Published:** 2019-11-22

**Authors:** Hao Wu, Dahai Dai, Xuesong Wang

**Affiliations:** State Key Laboratory of Complex Electromagnetic Environment Effects on Electronics and Information System, National University of Defense Technology, Changsha 410073, China; ddh1206@163.com (D.D.); wangxuesong@nudt.edu.cn (X.W.)

**Keywords:** radar, high-resolution range profile, principal component analysis, t-distributed stochastic neighbor embedding, density-based clustering

## Abstract

High-resolution range profile (HRRP) has attracted intensive attention from radar community because it is easy to acquire and analyze. However, most of the conventional algorithms require the prior information of targets, and they cannot process a large number of samples in real time. In this paper, a novel HRRP recognition method is proposed to classify unlabeled samples automatically where the number of categories is unknown. Firstly, with the preprocessing of HRRPs, we adopt principal component analysis (PCA) for dimensionality reduction of data. Afterwards, t-distributed stochastic neighbor embedding (t-SNE) with Barnes–Hut approximation is conducted for the visualization of high-dimensional data. It proves to reduce the dimensionality, which has significantly improved the computation speed. Finally, it is exhibited that the recognition performance with density-based clustering is superior to conventional algorithms under the condition of large azimuth angle ranges and low signal-to-noise ratio (SNR).

## 1. Introduction

As a device for transmitting and receiving electromagnetic waves, radar has played an indispensable role in in both civilian and military fields [1]. Radar automatic target recognition refers to the work of automatically identifying targets of interest with the acquired radar information [2,3]. In the field of radar target recognition, the main research objects can be divided into two categories: Natural and man-made objects. Natural targets mainly include lakes, mountains, trees, crops, etc., while man-made targets include typical targets such as buildings, billboards, airplanes, vehicles, unmanned aerial vehicles (UAVs), and ships. In the research of natural targets, many great achievements have been made [4,5,6,7]. However, the volume of the artificial target is relatively small compared to the natural target, and the structure is more complicated. Therefore, the difficulty for researchers has been correspondingly increased.

High-resolution range profile is the distribution of target scattering centers with radar echoes along the line of sight (LOS). The principle of radar high-resolution range profiles (HRRPs) is uncomplicated, which are liable to be acquired and analyzed, hence they have been widely studied. Many signal processing approaches have been developed in recent years. In reference [8], a novel detection and motion compensation algorithm has been proposed to solve the distortions of HRRPs when relative motions between the target and the radar happens. Aubry et al. have put forward a novel HRRP estimation algorithm that optimizes the probing waveforms and reduces the error according to the acquired information [9]. To reduce the effects of noise, an effective HRRP recognition approach can be obtained with the basis of orthogonal matching pursuit, which is validated by real-measured data [10]. Meanwhile, Guo et al. utilized one-dimensional residual-inception network to recognize HRRPs [11]. To improve the ability of recognizing HRRPs with bistatic radar, RELAX method and novel feature extraction processing have been used [12]. Nevertheless, the difficulties in recognizing HRRPs under the condition of low signal-to-noise ratio (SNR) have not been solved effectively.

In the area of HRRP recognition, various classification algorithms have been applied. An extended support vector data description (SVDD) method with negative examples has been proposed to classify four aircrafts exactly with a small number of training samples [13]. Pan et al. developed a method that contains discriminant deep belief network (DDBN) and statistic distribution analysis to recognize three kinds of aircraft [14]. With sparse representation classification criterion, a dictionary learning method has been utilized to improve the classification accuracy of aircrafts with different SNRs [15]. Feng et al. have built stacked autoencoder deep networks for feature extraction and recognition, which performs better than the shallow models [16]. Moreover, the multi-layer perception has been adopted to obtain the relationship between labels and samples with the demonstration of real-measured aircraft data [17]. Recent research on target classification mainly focus on supervised or semi-supervised learning, which needs the labels of targets that cannot be easily obtained in the real military field. Therefore, it is necessary to classify targets automatically without prior information. Unsupervised learning has been employed in feature extraction of HRRP samples in recent years [18,19,20,21,22]. In [18], a stacked autoencoder was utilized to learn features with different levels of data. A novel dictionary learning method has been adopted to extract robust features from HRRPs [19]. Ma et al. utilized stacked denoising and contractive encoder to study the hidden property of corner reflector HRRPs [20]. Local factor analysis [21] and denoising autoencoder [22] have been proposed by some other scholars to study features of HRRPs. Those unsupervised learning algorithms mainly focus on feature extraction, not for classification. As a consequence, we need to study the classification of HRRPs with unsupervised learning algorithms.

With the development of modern radar measurement technology, the data dimensionality of HRRPs has increased. Therefore, it is necessary to reduce the data dimensionality and improve processing ability. Principal component analysis (PCA) includes a procedure that transforms a number of correlated variables into a smaller number of uncorrelated variables called principal components [23,24]. Robust principal component analysis has been widely used by many researchers in radar application in recent years [25,26,27,28,29]. Robust PCA has been adopted to image rotating parts and estimate the rotating parameters with measured data [28]. Nguyen et al. used robust PCA to extract sources of interference from radar signals [29]. Visualization of high-dimensional data is able to effectively enhance the understanding of the distribution of target points, and is more conducive to human analysis and judgment, which can further reduce the target dimension and facility the auxiliary judgment. The t-distributed stochastic neighbor embedding (t-SNE) provides approaches to automatically obtain understanding from large datasets [30]. Most classification algorithms, including the recently popular deep learning, tend to obtain the labels of some specific samples in advance. However, in practical situations, especially in the battlefield, it is impossible to get the prior information of targets. The application of clustering algorithm is consequently indispensable [31,32]. In addition, a novel density-based clustering method can effectively improve the recognition performance of the target without training [33].

We study the HRRPs on basis of real-measured and electromagnetic calculation data. Firstly, we discuss the background of the issue and problems to be addressed. The signal model that includes the principle of HRRP formation is introduced in Section 2. Section 3 presents the proposed method, which is based on PCA, the accelerated t-SNE with Barnes–Hut approximation, and the density-based clustering algorithm. In Section 4, the experiment results are presented. Section 5 shows the conclusions of the paper and provides prospect for the future study.

The proposed algorithm contains three great advantages as mentioned below:

1. Effective and fast dimensional reduction. PCA greatly reduces the dimensionality of the data while preserving the HRRP information. Meanwhile, with the accelerated t-SNE, we can achieve further dimensionality reduction much faster than the conventional t-SNE.
2. Visualization of high-dimensional data. After the operation of PCA, the dimension of data is still high, and it is difficult to express the distribution of data points in 2D or 3D coordinate system. The t-SNE algorithm provides a valid approach to present data for visualization which is conducive to the intuitive judgment.
3. High accuracy of clustering without training. At this stage, many recognition algorithms need to be trained for classification. However, in some cases, especially in military field, the samples of specific targets cannot be obtained. In this paper, the high-accuracy HRRP clustering method can obtain classification results without training.

## 2. The Signal Model of HRRPs

The transmitted linear-frequency-modulated signals are defined as follows [34]

(1) $$s_{t}\left(t,\tau \right)=rect\left(\frac{\tau }{T_{p}}\right)\exp\left(j2\pi f_{c}t+j\pi \gamma \tau^{2}\right)$$

where τ is fast time, t is full time; rect(⋅) is the operation of rectangular window and the width is defined as $T_{p}$; carrier frequency is $f_{c}$ and the chirp rate is γ. 

When the wavelength of radar is much smaller than the measured target, the scattering model can be simplified as scattering centers in high-frequency regime. We suppose that the target consists of M scattering points.

We define the distance from the scatter points to radar as

(2) $$R_{i}\left(t\right),\;\mathrm{where}\;i\in \left[1,M\right],i\in N.$$

The returned echoes with scattering point model are represented as

(3) $$\begin{array}{ll} s_{r}\left(t,\tau \right)=\sum_{i=1}^{M} & A_{i}rect\left(\frac{\tau -\frac{2R_{i}\left(t\right)}{c}}{T_{p}}\right)\exp\left(j2\pi \left(f_{c}t-f_{c}\frac{2R_{i}\left(t\right)}{c}\right)\right)\\ & \times \exp\left(j\pi \gamma {\left(\tau -\frac{2R_{i}\left(t\right)}{c}\right)}^{2}\right) \end{array}$$

where $A_{i}$ denotes amplitude, which is related to the radar cross section, and c is the speed of light and the slow time is $t_{n}=t-\tau$.

After the operations of dechirping and phase compensation, the returned echoes can be written as

(4) $$\begin{array}{ll} s_{r1}\left(t,\tau \right)= & \sum_{i=1}^{M}A_{i}rect\left(\frac{\tau -\frac{2R_{i}\left(t_{n}\right)}{c}}{T_{p}}\right)\exp\left(j\frac{4\pi }{c}R_{\Delta i}\left(t_{n}\right)\cdot f_{c}\right)\\ & \times \exp\left(j\frac{4\pi }{c}R_{\Delta i}\left(t_{n}\right)\cdot \gamma \left(\tau -\frac{2R_{ref}\left(t_{n}\right)}{c}\right)\right) \end{array}$$

where $R_{ref}\left(t_{n}\right)$ is the reference distance in the dechirping progress at the slow time $t_{n}$, and $R_{\Delta i}\left(t_{n}\right)=R_{p}\left(t_{n}\right)-R_{ref}\left(t_{n}\right)$. We perform the Fourier transform to Equation (4) along the fast time dimension, and the HRRPs can be shown as

(5) $$s_{r2}\left(r,t_{n}\right)=\left|\sum_{i=1}^{M}A_{i}\exp\left(j4\pi R_{\Delta i}\left(t_{n}\right)/\lambda_{c}\right)\mathrm{sinc}\left(\frac{2B}{c}\left(r-R_{\Delta i}\left(t_{n}\right)\right)\right)\right|$$

where B is the bandwidth and $\lambda_{c}=c/f_{c}$.

## 3. Proposed Method

### 3.1. Principal Component Analysis Based on Singular Value Decomposition

Principal component analysis (PCA) is a predominant algorithm for dimensionality reduction and extraction of independent elements in mathematical statistics [24]. It contains eigenvalue decomposition, singular value decomposition, and generalized singular value decomposition, etc. In order to effectively remove the redundancy of HRRPs and increase the calculation speed, it is necessary to extract the subspace features by principal component analysis. In this paper, aiming to minimize the loss of features, we utilize singular value decomposition method to achieve the effect of data dimensionality reduction.

We define the matrix D that represent the data sets of HRRPs, where the rows correspond to observations with different azimuth angles of targets and the columns correspond to the absolute values of amplitudes along the range of the line of sight. With SVD, the data matrix can be divided into three parts, shown as follows:

(6) $$D=P\Sigma Q^{T}$$

where P denotes the eigenvectors of matrix $DD^{T}$ and Q represent the eigenvectors of matrix $D^{T}D$. The columns of P and Q are the right and left singular vectors of the data matrix. The diagonal elements of matrix $\Sigma^{2}$ are both the eigenvalues of $DD^{T}$ and $D^{T}D$.

Then, reconstruct the data matrix by selecting the principal component, which is denoted as:

(7) $$\tilde{D}=\tilde{P}\tilde{\Sigma }{\tilde{Q}}^{T}$$

where the size of reconstructed matrix $\tilde{D}$ is M by $N_{r}$ (where $N_{r}<N$) and $N_{r}$, which can preserve features of data, is the number of eigenvalues selected from the diagonal matrix with descending order. The columns of $\tilde{P}$ and $\tilde{Q}$ correspond to the eigenvalues of $\tilde{D}$. 

The definition of eigenvalue ratio $r_{e}$ is given by

(8) $$r_{e}=\frac{\sum_{l=1}^{N_{r}}\lambda_{l}}{\sum_{l=1}^{N}\lambda_{l}}$$

where $\lambda_{l}$ is the corresponding eigenvalue. More details can be found in reference [24].

### 3.2. Accelerated t-SNE with Barnes–Hut Approximation 

T-distributed stochastic neighbor embedding (t-SNE) is a valid method to project the $N_{r}$-dimensional HRRP into the c-dimensional (c is much smaller than $N_{r}$) embedding and it is convenient to analyze low-dimensional data for visualization [30].

The dataset of HRRP is $X=\left\{x_{1},x_{2},x_{3},\cdots ,x_{i},\cdots ,x_{M}\right\},X_{i}\in R^{N_{r}}$ and after the transformation of t-SNE HRRPs can be represented as $Y=\left\{y_{1},y_{2},y_{3},\cdots ,y_{i},\cdots ,y_{M}\right\},y_{i}\in R^{c}$.

The conditional probabilities of similarity between input HRRPs are expressed as

(9) $$p_{j|i}=\{\begin{array}{c} \frac{\exp\left(-{\left\|x_{j}-x_{i}\right\|}^{2}/2\sigma_{i}^{2}\right)}{\sum_{k\neq i}\exp\left(-{\left\|x_{k}-x_{i}\right\|}^{2}/2\sigma_{i}^{2}\right)},i\neq j\\ \;0\;,\;i=j \end{array}$$

Joint probabilities $p_{ij}$, which measure the pairwise similarity between the HRRP samples $x_{i}$ and $x_{j}$, are denoted as

(10) $$p_{ij}=\frac{p_{j|i}+p_{i|j}}{2M}$$

We utilize the normalized student-t to indicate the relationship between the output $y_{i}$ and $y_{j}$ in the projection embedding with a single degree of freedom:

(11) $$q_{ij}=\{\begin{array}{c} \frac{{\left({\left\|y_{j}-y_{i}\right\|}_{2}^{2}+1\right)}^{-1}}{\sum_{k\neq i}{\left({\left\|y_{k}-y_{i}\right\|}_{2}^{2}+1\right)}^{-1}},i\neq j\\ \;0\;,\;i=j \end{array}$$

The Kullback–Leibler divergence between input and output probability distribution is

(12) $$C=\sum_{i\neq j}p_{ij}\log\frac{p_{ij}}{q_{ij}}$$

In order to minimize the value of Equation (12), the gradient is given by

(13) $$\frac{\partial C}{\partial y_{i}}=4\sum_{j}\left(q_{ij}-p_{ij}\right)\left(y_{j}-y_{i}\right){\left({\left\|y_{i}-y_{j}\right\|}_{2}^{2}+1\right)}^{-1}$$

However, the computation resource is obviously limited due to the scale of t-SNE algorithm varies quadratically with the number of total samples M. Due to the limitation of time and calculation capability, the maximum number of samples is below 10,000 in ordinary application and the real-time condition cannot be satisfied.

The gradient can be divided into the attractive and repulsive forces [35]. Barnes–Hut approximation is an effective algorithm to reduce the calculation burden brought by the repulsive forces. It is based on the theory of quadtree, which consists of nodes that indicates a cell with different size parameters. We minimize Equation (12) with Barnes–Hut approximation to obtain the optimal projection datasets $Y^{*}={\left\{y_{i}^{*}\right\}}_{i=1}^{M},y_{i}^{*}\in R^{c}$.

(14) $$Y^{*}=\arg\underset{Y}{\min}\sum_{i\neq j}p_{ij}\log\frac{p_{ij}}{q_{ij}}$$

### 3.3. A Novel Density-Based Clustering 

The clustering algorithm is able to classify objects automatically with no prior information in unsupervised learning. A novel density-based clustering approach focuses on the property that the density values of neighbors around the cluster centers are much lower than that of the cluster centers and the distances between cluster centers are relatively large. It has been proved to be robust and effective on several different datasets [33]. In this algorithm, local density $\rho_{i}$ and distance from higher-density point $\delta_{i}$ are the key parameters that show the property of objects and the subscript i is the data point. 

The local density can be represented by the cutoff or gaussian kernel. The definition of cut-off kernel is given by

(15) $$\rho_{i}=\sum_{j\neq i}\chi \left(d_{ij}-d_{c}\right)$$

where

(16) $$\chi \left(x\right)=\{\begin{array}{c} 0,\;x\geq 0\\ 1,\;x<0 \end{array}$$

$d_{c}$ denotes the cutoff distance and $d_{ij}$ represents the distance between points i and j. 

Suppose there are $N_{1}$ points in the dataset $I_{S}$; the distance is defined as follows

(17) $$\delta_{q_{i}}=\{\begin{array}{ll} \underset{j\geq 2}{\max}\left\{d_{q_{i}}d_{q_{j}}\right\},\; & i=1\\ \underset{j<i}{\min}\left\{\delta_{q_{j}}\right\}\;,\; & i\geq 2 \end{array}$$

where $\rho_{q_{1}}\geq \rho_{q_{2}}\cdots \geq \rho_{q_{i}}\cdots \geq \rho_{q_{N_{1}}}$. 

The simple version of the novel density-based algorithm, which contains five steps, is listed in Algorithm 1.

**Algorithm 1.** Simple version of the novel density-based algorithm. The steps for the novel density-based algorithm.1: Input: Distance $d_{ij},i<j,i,j\in I_{S}$2: Initialization: Cutoff distance $d_{c}=d_{\left[\frac{1}{2}N_{1}\left(N_{1}-1\right)t+\frac{1}{2}\right]}$, where [] represent rounding function and 0.1≤t≤0.2. Attribute of points $n_{i}=0,i\in I_{S}$.3: Results: Number of categories of the HRRPs and the type of each sample.4: Begin5: Step 1. The computation of ${\left\{\rho_{i}\right\}}_{i=1}^{N_{1}}$ and ${\left\{q_{i}\right\}}_{i=1}^{N_{1}}$.6: Step 2. Calculation of distance and attribute ${\left\{n_{i}\right\}}_{i=1}^{N_{1}}$.7: Step 3. Identification of clustering centers and classification of the other points.8: Step 4. The selection of average local density in the border region.9: Step 5. Classification of points with the label of cluster core or cluster halo.10: End

### 3.4. Overall Structure of Proposed Method

**Figure 1:**
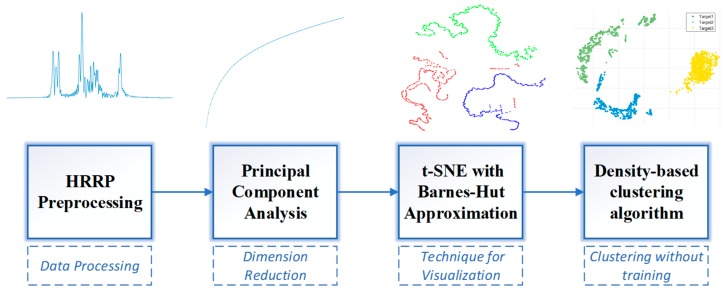
The structure of proposed method.

The overall structure of our proposed method is shown in Figure 1. Firstly, we employ the absolute value of the HRRP amplitude for further dimension reduction and recognition. Secondly, we utilize principle component analysis to reduce the dimension of HRRP data, which can subtract data redundancy and reduce processing time. Visualization of high dimensional data provides people with an intuitive understanding and it also compresses HRRPs effectively. Thirdly, we adopt the algorithm of t-SNE with Barnes–Hut approximation, which is much faster than the conventional t-SNE. Finally, most classification algorithms in radar automatic target recognition need to obtain target labels in advance, while the attribute information of targets cannot be acquired under some specific conditions. Therefore, a predominant clustering algorithm is demanded, and we take advantage of a novel density-based clustering algorithm to accomplish accurate classification of man-made objects without training.

## 4. Experiment Results

**Figure 2:**
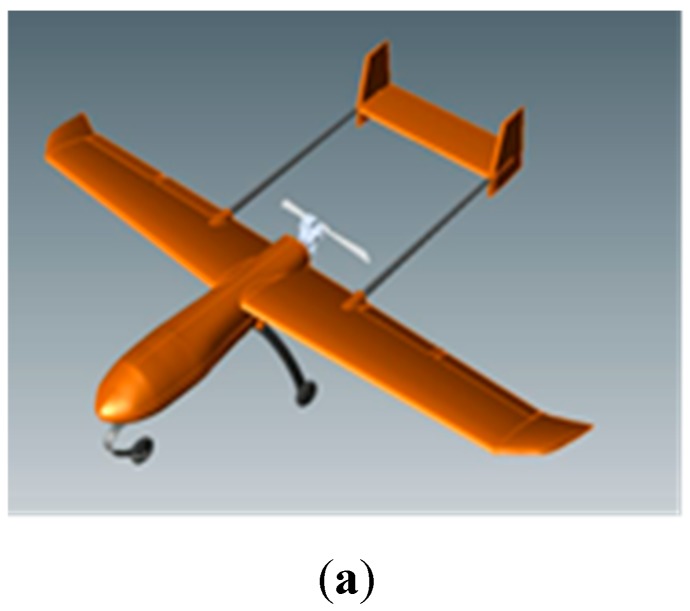

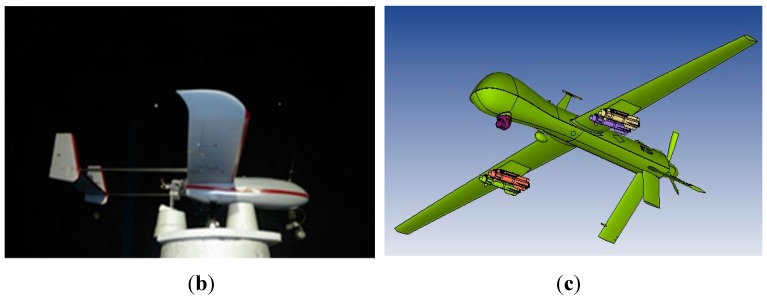
Real-measured or electromagnetic calculation models of three unmanned aerial vehicles (UAVs); (**a**) UAV1; (**b**) UAV2; (**c**) UAV3.

We firstly focus on three types of UAV targets, namely UAV1, UAV2, and UAV3. UAV1 and UAV2 have the same structure and size with different material. Data of UAV1 and UAV3 are calculated by electromagnetic software, while UAV2 has been measured in anechoic chamber. The pith angle of the measurement is 0° and the azimuth angle range in this paper is 60°. The actual pictures or electromagnetic calculation models of these three types of UAV targets are shown in Figure 2. The frequency ranges from 8 to 12 GHz with the interval of 20 MHz and all data are acquired with full polarization. All experiments were performed on a PC with a 3.20 GHz i7-87000 CPU and 16 GB RAM.

**Figure 3:**
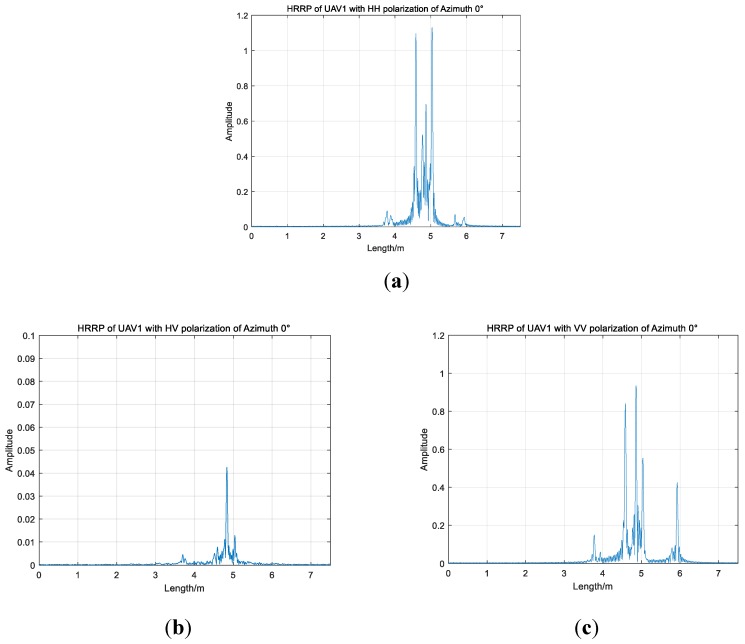
High-resolution range profile (HRRPs) of UAV1 with full polarization of azimuth 0°; (**a**) UAV1 of HH channel; (**b**) UAV1 of HV channel; (**c**) UAV1 of VV channel.

HRRPs of UAV1 with full polarization of azimuth 0° are presented in Figure 3. HH, HV and VV are three polarization channels. The average amplitudes of HH and VV channels are much larger than that of the HV channel. However, the amplitude distributions of UAV HRRPs with HH and VV polarization are roughly similar. Strong scattering points are concentrated in the range from 3.5 to 6 m. Between 4.4 m and 5.2 m, the energy of the one-dimensional range image is obviously concentrated, and more than three peaks appear with HH and VV polarization. When the azimuth angle is 0°, the positions and numbers of strong scatters are approximately identical to co-polarized channels. However, comparing HH and VV channels, the relative magnitudes of the same channel are different according to Figure 3a,c. With high-resolution range profiles of three channels, we are able to estimate the approximate size information roughly.

**Figure 4:**
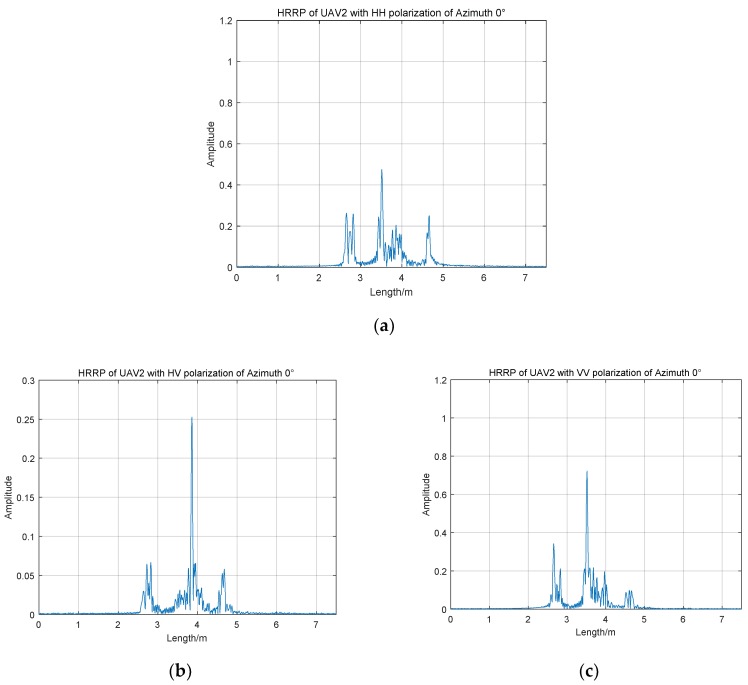
HRRPs of UAV2 with full polarization of azimuth 0°; (**a**) UAV2 of HH channel; (**b**) UAV2 of HV channel; (**c**) UAV3 of VV channel.

Figure 4 displays the HRRPs of UAV2 with full polarization at the azimuth angle of 0 degrees. In contrast with UAV1 with the same azimuth angle, the data of UAV2, which is real measured in an anechoic chamber, shows smaller amplitude differences among three polarization channels. Most energy of the three HRRPs is centralized between the range of 2.5 to 5 m and each channel has a maximum amplitude that indicates a strong scattering center between 3.5 and 4 m. Except for the strong scattering center, the amplitude distribution in other range areas is relatively uniform. HRRPs of UAV2 with HH and VV polarization are weaker than those of UAV1 at the same azimuth angle of 0°. Nevertheless, the real-measured data of UAV2 in HV channel retain higher amplitudes.

**Figure 5:**
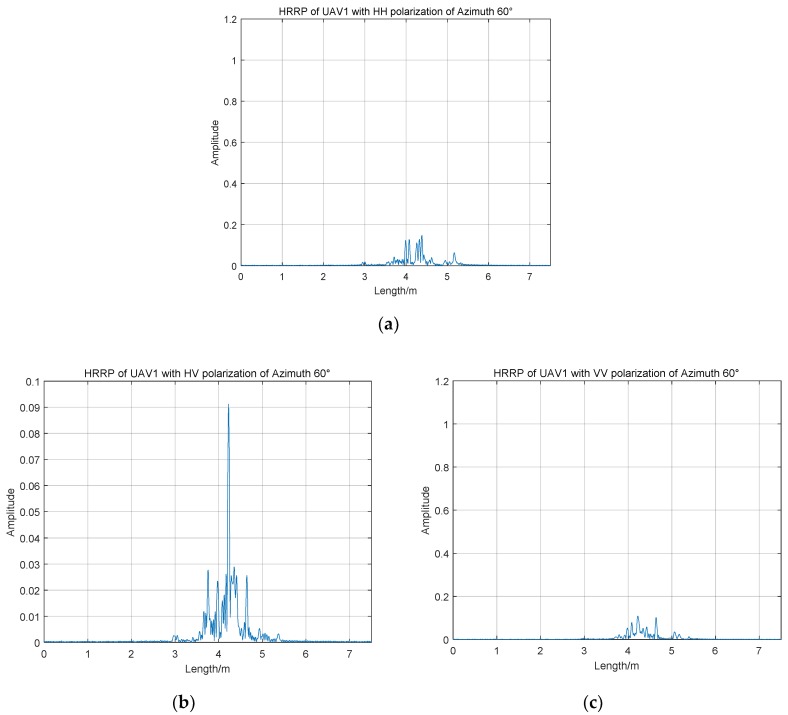
HRRPs of UAV1 with full polarization of azimuth 60°; (**a**) UAV1 of HH channel; (**b**) UAV1 of HV channel; (**c**) UAV1 of VV channel.

As shown in Figure 5, HRRPs of UAV1 of three polarization channels of azimuth 60° are different from Figure 3. It is apparent that amplitudes of range profiles are smaller than those of the azimuth angle of 0 degrees in HH and VV polarization channels. The maximum amplitudes of the co-polarized HRRPs are both less than 0.2 and there is no range cell with a particularly high amplitude value. There is a significant difference between the amplitudes of HRRPs in HV polarization channel with azimuth angles of 0° and 60°, and the average amplitude of azimuth 0 degrees is higher than that of 60°, which is different from those of HH and VV polarization channel. Therefore, it is necessary for us to analyze the mean amplitude at different azimuth angles.

**Figure 6:**
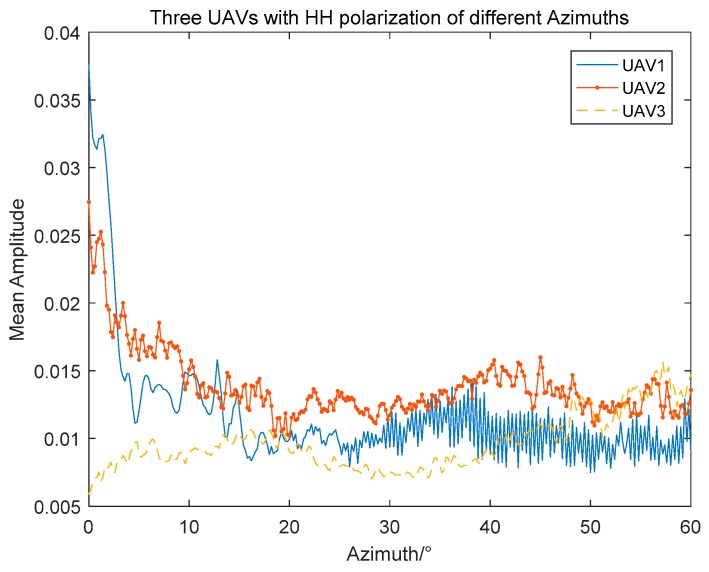
HRRPs of three UAVs at different azimuth angles with HH polarization.

As shown in Figure 6, the fluctuation for UAV1 and UAV2 in mean amplitudes is approximately consistent from azimuth 0 to 60 degrees, because they are identical in structure and size. The mean amplitudes of UAV3 are mostly inferior to the other two UAVs in the azimuth angle range from 0 to 40°. When the azimuth angle scope is between 30° and 60°, the mean amplitude of UAV3 shows an uptick in volatility. In the same azimuth angle range, the amplitude changes of UAV 1 and UAV 2 are approximately the same, and the mean amplitude of UAV1 is greater than UAV2 in most cases due to the differences of shape and material properties. It is difficult to identify different UAVs with the average amplitude. Therefore, the amplitude information of HRRPs should be deeply studied.

**Figure 7:**
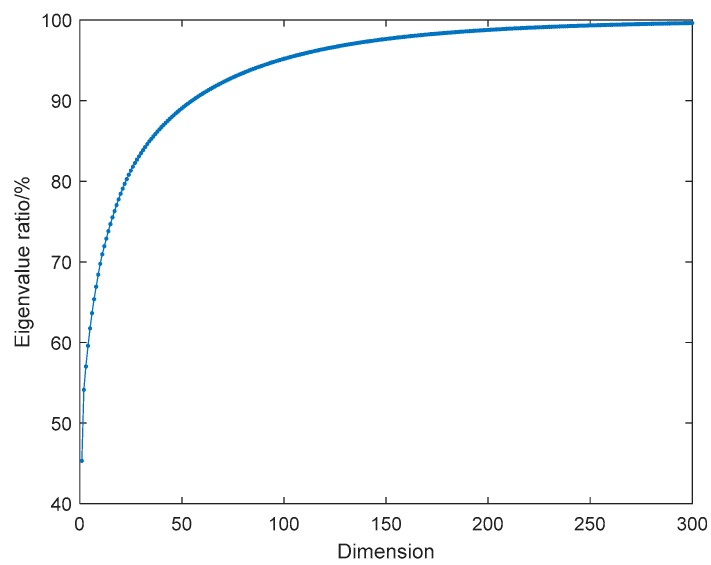
Eigenvalue ratio of three UAVs with principal component analysis (PCA).

We choose the HRRPs of HH polarization channel to carry out the study. Since the dimension of each sample is 1024, it is necessary to reduce the dimension and remove the data redundancy through principal component analysis. However, the selection of dimension size is a significant issue. The goal to reduce the target dimension as much as possible with the amplitude information of HRRPs well preserved should be achieved. The information loss of HRRP data can be obtained according to the eigenvalue ratio. As can be seen from the Figure 7, as the dimension increases, the eigenvalue ratio gradually increases, and finally approaches 100%. When the dimension is chosen to be 300, the eigenvalue ratio exceeds 99.5%, and there is almost no loss of information of HRRPs. Therefore, in this paper, we select 300 as the dimension size in PCA processing and provide the processed HRRP data to the subsequent accelerated t-SNE for further dimension reduction and visualization.

**Figure 8:**
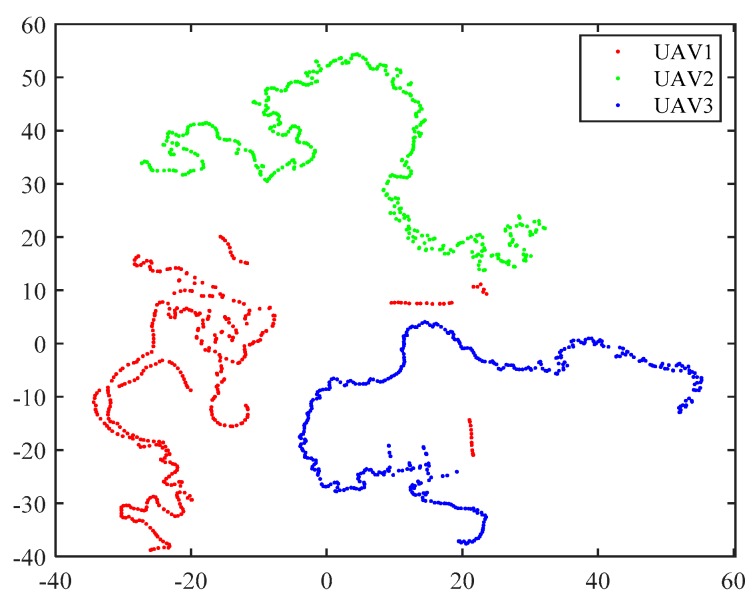
Visualization of three UAVs by t-distributed stochastic neighbor embedding (t-SNE) technique (with Barnes–Hut algorithm) in 2D coordinate system.

T-SNE is an effective high-dimensional data visualization algorithm. It can be utilized to intuitively obtain the distribution of UAV data points. In addition, it is also a data dimension reduction algorithm, which reduces the computational burden for subsequent classification work. The traditional t-SNE algorithm is inefficient in calculation. By adopting the Barnes–Hut algorithm, it is possible to quickly obtain data in 2D, 3D, and other multidimensional conditions. Visualization of data points of three types of UAV targets in two-dimensional coordinate systems is given in Figure 8. In Figure 8, a small number of data points of UAV1 are close to those of UAV3, which may cause difficulties in clustering. In this paper, we set the output dimension of each HRRP sample to be 5 after the t-SNE algorithm with Barnes–Hut processing.

**Figure 9:**
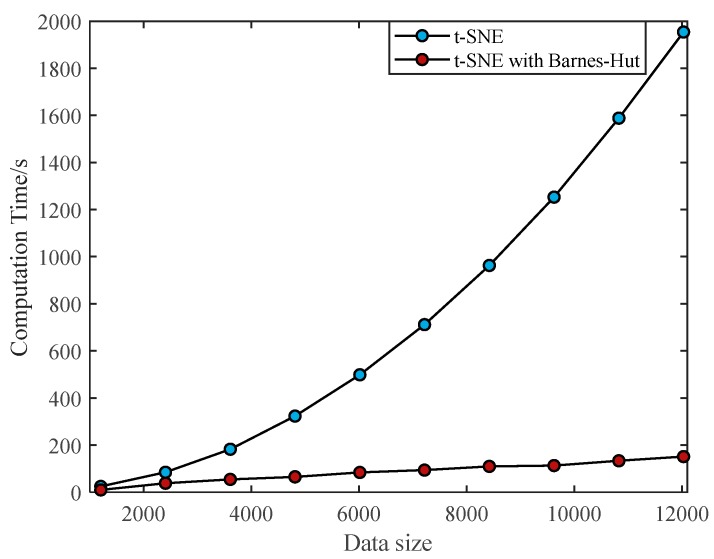
Computation time between conventional t-SNE and accelerated t-SNE with Barnes–Hut algorithm with different data sizes.

Figure 9 shows the comparison of the calculation time of traditional t-SNE and accelerated t-SNE with Barnes–Hut algorithm under different sample sizes. In this experiment, the UAV HRRPs containing noise is generated by Monte Carlo simulation, thus a large number of HRRP samples are provided with the same SNR. As can be seen from Figure 9, with Barnes–Hut algorithm, we can effectively reduce the computation time. As the samples of HRRPs increase, the calculation time of conventional t-SNE increases exponentially, while the calculation time of t-SNE using the Barnes–Hut algorithm arises slowly and linearly. The larger the amount of data to be processed, the more prominent time advantage will be using the Barnes–Hut algorithm, which enhances the capability of real-time data processing.

**Figure 10:**
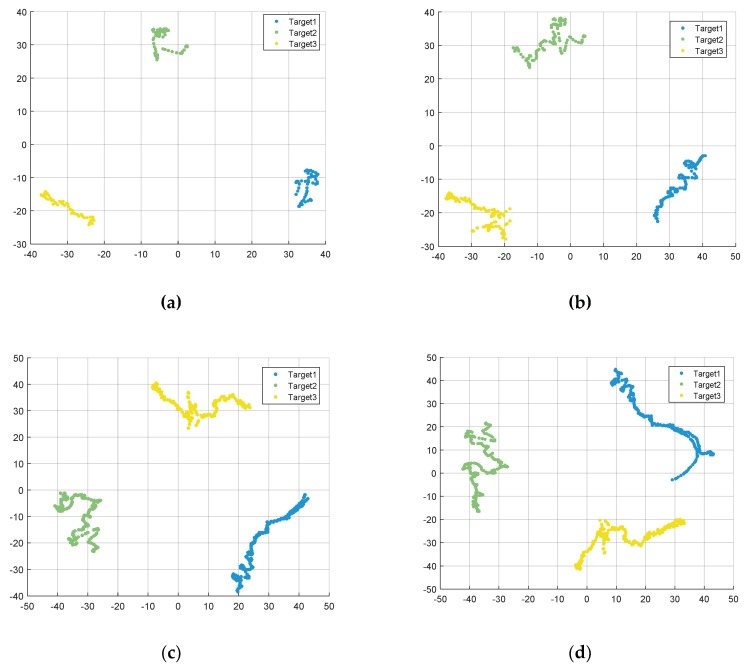

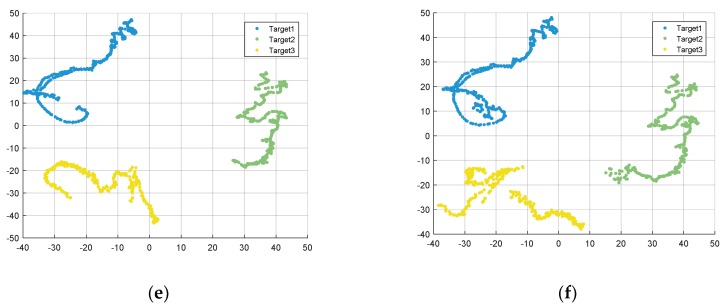
Clustering results of three UAVs with the signal-to-noise ratio (SNR) of 40dB with different azimuth angle ranges. (**a**) Azimuth angle range of 10°; (**b**) azimuth angle range of 20°; (**c**) azimuth angle range of 30°; (**d**) azimuth angle range of 40°; (**e**) azimuth angle range of 50°; (**f**) azimuth angle range of 60°.

In order to clearly show the difference in the run-time brought by the two algorithms clearly, we select the last set of data in Figure 10. We could see in Table 1 that the total calculation time of conventional t-SNE algorithm is close to 2000 s, while it takes less than 151 s using the Barnes–Hut algorithm, which has improved the processing efficiency by an order of magnitude. To further demonstrate the real-time processing of HRRP data, we give the average execution time of samples. The conventional algorithm takes about 0.16 s, while the proposed algorithm only needs even less than 0.013 s. This algorithm significantly improves the real-time visualization and dimensional reduction ability of UAV HRRPs.

As can be seen from Figure 10, when the SNR is 40 dB, the proposed algorithm can provide 100% classification accuracy under six different azimuth angles from 10° to 60° with the interval of 10°. After the processing of t-SNE with Barnes–Hut algorithm, we utilize the density-based and fast search clustering algorithm to separate the three types of UAV HRRP samples in the clustering space. In the case of different azimuth angle ranges, the classification results show robustness under the condition of high SNR. Although the classification accuracy of the HRRPs has been high enough under the condition of high SNR, it is still necessary to study the clustering under the condition of low SNR to further prove the superiority of the proposed algorithm.

**Figure 11:**
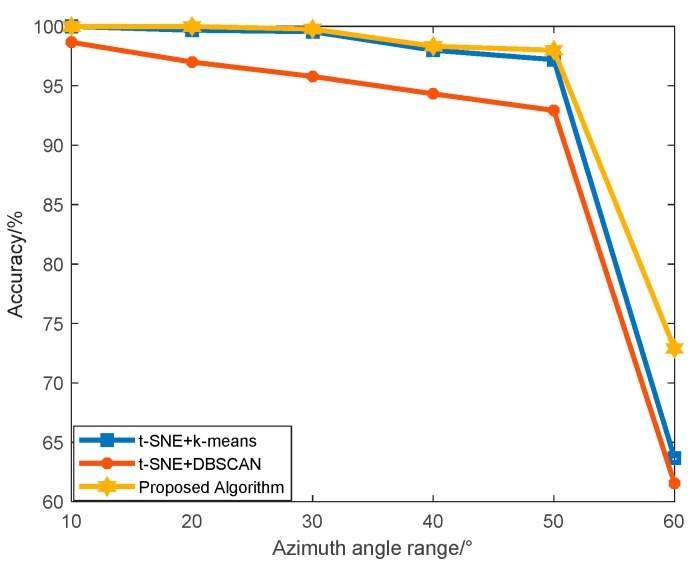
Classification results of three UAVs with different azimuth angle ranges between the proposed algorithm and conventional algorithms with the SNR of 5 dB.

As shown in Figure 11, when the SNR is 5 dB, the classification results of different algorithms are worse than that of 40 dB. The effects of noise and azimuth range on the algorithm are also clearer. With the increase of azimuth angle range, the accuracy of classification of the three algorithms decreases. The clustering accuracy of our proposed algorithm is superior to two conventional methods, which include k-means and DBSCAN with different azimuth angle ranges. Since the clustering algorithm cannot obtain any label information of the targets, it is easy to generate confusion under the condition of large azimuth angle range and low SNR. Under the condition that the azimuth angle range is 60° and the SNR is 5 dB, the accuracy of classification with conventional algorithms are both inferior to 64%. However, the algorithm that we have proposed in this paper can achieve an accuracy of nearly 73%, and it still maintains a high accuracy under low SNR and large azimuth angle range, which fully demonstrates superiority and reliability of the proposed algorithm.

**Figure 12:**
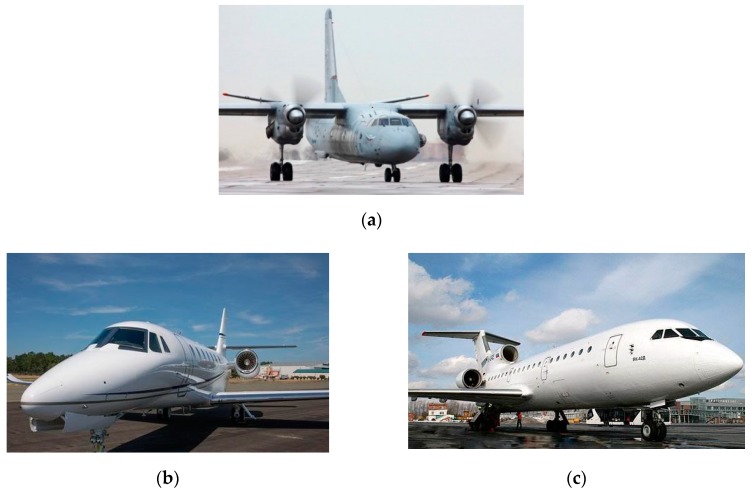
Three imagery planes; (**a**) An-26; (**b**) Cessna Citation; (**c**) Yark-42.

In order to further verify the reliability of the proposed algorithm, we utilized the real-measured HRRPs from three different kinds of flying planes, which contain the An-26, the Cessna Citation, and the Yark-42. The models of three planes are shown in Figure 12, where Figure 12a–c represents the An-26, the Cessna Citation, and the Yark-42, respectively. The An-26 is a propeller plane with medium size, the Cessna Citation is a jet plane with small size, and the Yark-42 is a large-sized jet plane. The parameters of planes in the experiment are shown in Table 2. All the HRRPs in this experiment are measured by a C-band radar system, which ranges 5.32–5.72 GHz. The wavelength of radar is 0.05 m and the pulse repetition frequency is 400 Hz. The radar transmits the chirp signal and the range resolution is 0.375 m. 

**Figure 13:**
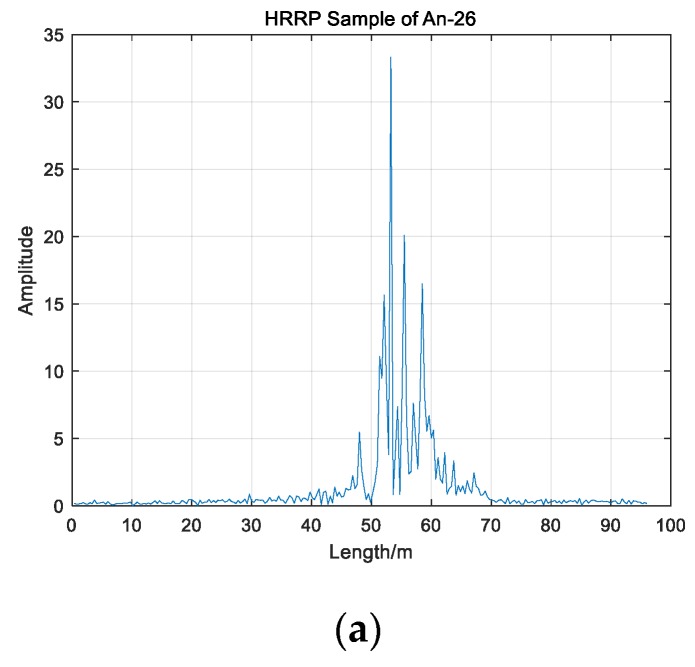

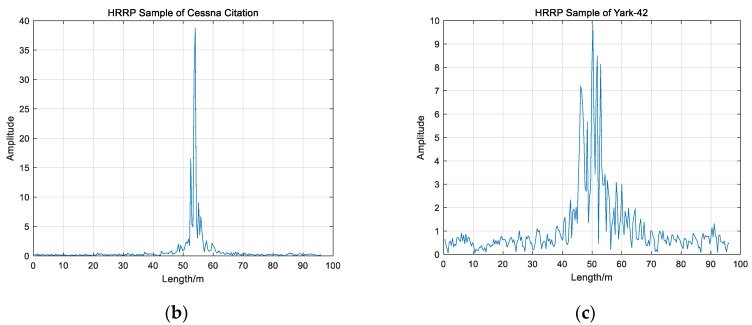
HRRP samples of three planes; (**a**) An-26; (**b**) Cessna Citation; (**c**) Yark-42.

Figure 13 shows HRRP samples of three planes in this experiment. It can be seen from Figure 13 that there are differences in HRRPs of three planes. HRRPs contains the size, structure, material, and other information between different planes. Therefore, the study on the difference of HRRPs is conducive to the automatic recognition of radar target. In this paper, we choose 1000 HRRP samples of each plane; namely, 3000 samples have been utilized to test the performance of three different algorithms. The number of range elements of all HRRP samples is 256; that is, each HRRP is a 256-dimensional vector in this experiment. 

**Figure 14:**
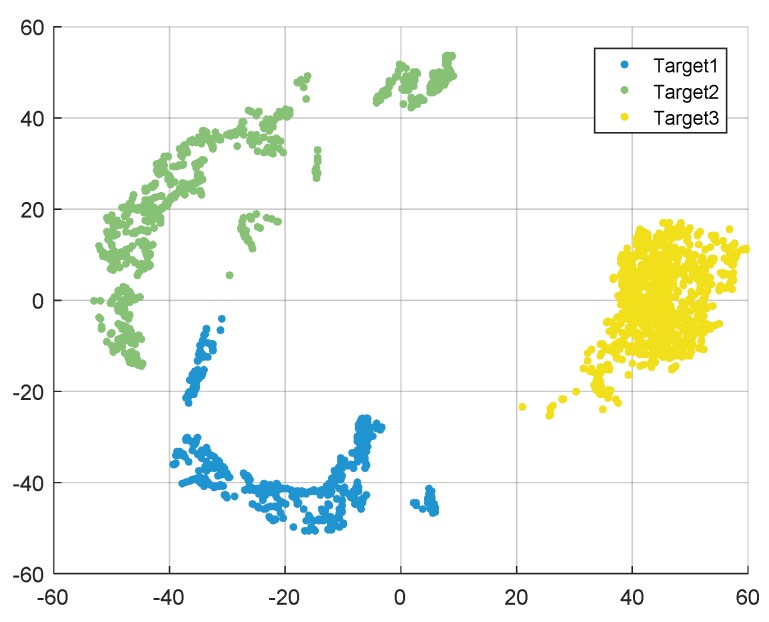
Clustering results of three flying planes with the proposed algorithm.

As shown in Figure 14, the points with three different colors represent planes after classification. With the proposed algorithm, all three planes are clustered automatically with high classification accuracy. It can be seen from Table 3 that after adopting accelerated t-SNE, the classification accuracy of k-means and DBSCAN is 92.17% and 92.00%, respectively. However, the clustering accuracy of the proposed algorithm is higher than that of the other two conventional algorithms, which reaches 94.23%. The validity and robustness of the proposed algorithm have been proved in different datasets. The algorithm not only improves the calculation speed and reduces the amount of data, but also effectively enhances the accuracy of classification and provides more possibilities for automatic real-time target recognition.

## 5. Conclusions

This study sets out to recognize targets automatically based on high-dimensional data reduction and visualization with HRRPs. On the basis of analyzing and preprocessing of HRRPs, we utilize principal component analysis to reduce the dimensionality. Furthermore, we adopt t-SNE with Barnes–Hut approximation to carry out the visualization of HRRPs and further data dimensionality reduction with higher processing speed. We cluster HRRPs without labels more accurately than other conventional algorithms.

The research of HRRPs is definitely a frontier issue. The methods that effectively extract, detect, and identify objects in the complex electromagnetic environments such as ground clutter and sea clutter are still urgent to be found. In the later stage, an in-depth study will be carried out on the recognition of targets in the harsh clutter condition, so as to truly realize the reliable identification of targets in different environments with HRRPs.

## Figures and Tables

**Table 1:** The run-time of two algorithms.

Algorithm	Total Time(s)	Average Time(s)
Conventional t-SNE	1954.156	0.1617
Accelerated t-SNE with Barnes–Hut	150.771	0.0125

**Table 2:** Parameters of planes in the experiment.

Aircraft	Length/m	Width/m	Height/m
An-26	14.40	15.90	4.57
Cessna Citation	23.80	29.20	9.83
Yark-42	36.38	34.88	9.83

**Table 3:** Classification accuracy of different algorithms with HRRP samples.

Algorithm	Accelerated t-SNE + k-Means	Accelerated t-SNE + DBSCAN	Proposed Algorithm
Accuracy	92.17%	92.00%	94.23%
